# The complete chloroplast genome sequence of *Polyalthiopsis verrucipes* (Annonaceae)

**DOI:** 10.1080/23802359.2021.1942258

**Published:** 2021-10-20

**Authors:** Hui Zhang, Chen Xiu, Sheng Zhou, Ping Chen, Yi Wang

**Affiliations:** College of Horticulture and Landscape Architecture, Zhongkai University of Agriculture and Engineering, Guangdong, China

**Keywords:** *Polyalthiopsis verrucipes*, complete cp genome, automated assembly

## Abstract

*Polyalthopsis* Chaowasku is a recently newly described genus in Annonaceae. *Polyalthopsis verrucipes* (C.Y.Wu ex P.T.Li) B.Xue & Y.H.Tan is distributed in Southern Yunnan, China. In this article, we report the complete chloroplast (cp) genome of *Polyalthiopsis* based on Illumina sequencing data. The whole cp genome of this species is 159,965 bp in length, consisting of two inverted repeat regions (IR, 25,974 bp each), one large single-copy region (LSC, 89,030 bp), and one small single-copy region (SSC, 18,987 bp). A total of 130 genes were annotated for the cp genome, including 83 protein-coding genes, 37 tRNAs, and 8 rRNAs. Phylogenetic analysis indicated that *P. verrucipes* was closely related to *Meiogyne hainanensis.*

The genus *Polyalthiopsis* Chaowasku is a recently published small genus in Annonaceae (Chaowasku, Damthongdee, et al. 2018; Xue et al. 2020). It includes one species in Vietnam and two species in China (Xue et al. 2020). It belongs to the tribe Miliuseae, and is sister to the genus *Miliusa* Lesch. ex A. DC. (Xue et al. 2020). The long-recognized sister relationship between *Miliusa* and *Huberantha* in previous phylogenetic studies (e.g. Chaowasku, Thomas, et al. 2014) has been redefined following the inclusion of *Polyalthiopsis*.

*Polyalthiopsis verrucipes* (C.Y.Wu ex P.T.Li) B.Xue & Y.H.Tan is distributed in Menghai, Yunnan, with restricted distribution and small populations. It was transferred from the genus *Polyalthia* Blume (Xue et al. 2020). This species is only represented by very few collections, and it is considered to be critically endangered as the habitat is severely destroyed (Xue et al. 2020).

In this study, we reported the complete chloroplast (cp) genome sequence of *P. verrucipes*, which represents the first plastid genome in the genus and the data would be helpful for the phylogenetic study of *Polyalthiopsis* and other closely related genera in the future.

The fresh leaves of *Polyalthopsis verrucipes* were collected from Menghai, Xishuangbanna, Yunnan province, China (21°46′22.5′′N, 100°02′58.5′′E). The total genomic DNA of the species was extracted using the modified CTAB method (Doyle and Doyle 1987). A voucher specimen (*B. Xue & H.B. Ding XB311*) was deposited in the herbarium of South China Botanical Garden, Chinese Academy of Sciences (IBSC) with the barcode number IBSC0860085. Library construction and sequencing were performed by BGI-Shenzhen (Shenzhen, China), using an Illumina HisSeq 2500 Sequencing System following the manufacturer’s instructions. The cp genome was assembled by the GetOrganelle (Jin et al. 2020). Using *Meiogyne hainanensis* (NC_043867.1) (recorded as *Chieniodendron hainanense* in Genbank) as a reference, the annotations were implemented using the online software Geseq (Tillich et al. 2017) with final manually correction. Finally, the complete sequences and annotations of *P. verrucipes* were submitted to GenBank with the accession number MW018366.

The complete cp genome of *P. verrucipes* is 159,965 bp in length, containing a large single-copy (LSC) region of 89,030 bp, a small single-copy (SSC) region of 18,987 bp, and two inverted repeat (IR) regions of 25,974 bp each. The whole cp genome has 130 genes in total, including 85 protein-coding genes, 37 tRNA genes, and 8 rRNA genes. In addition, the overall GC content of the genome is 39.05%. In order to investigate the phylogenetic relationships of *P. verrucipes* with other species in Annonaceae and outgroup species, another two complete cp genomes from Annonaceae and 11 complete cp genomes from several closely related families Aristolochiaceae, Magnoliaceae, Myristicaceae, and Lauraceae were obtained from GenBank and aligned with *P. verrucipes* using MAFFT (Katoh and Standley 2013). A maximum likelihood analysis was performed by RAxML (Stamatakis 2014) under GTR + G model with 1000 bootstrap replicates. *Aristolochia macrophylla* (GenBank Accession NC_041453.1) and *Aristolochia tubiflora* (Genbank Accession NC_041456.1) were selected as outgroups. The phylogeny showed that *P. verrucipes* was closely related to genus *Meiogyne* in Annonaceae; the family Annonaceae is closely related to Magnoliaceae and Myristicaceae (Figure 1).

**Figure 1.**
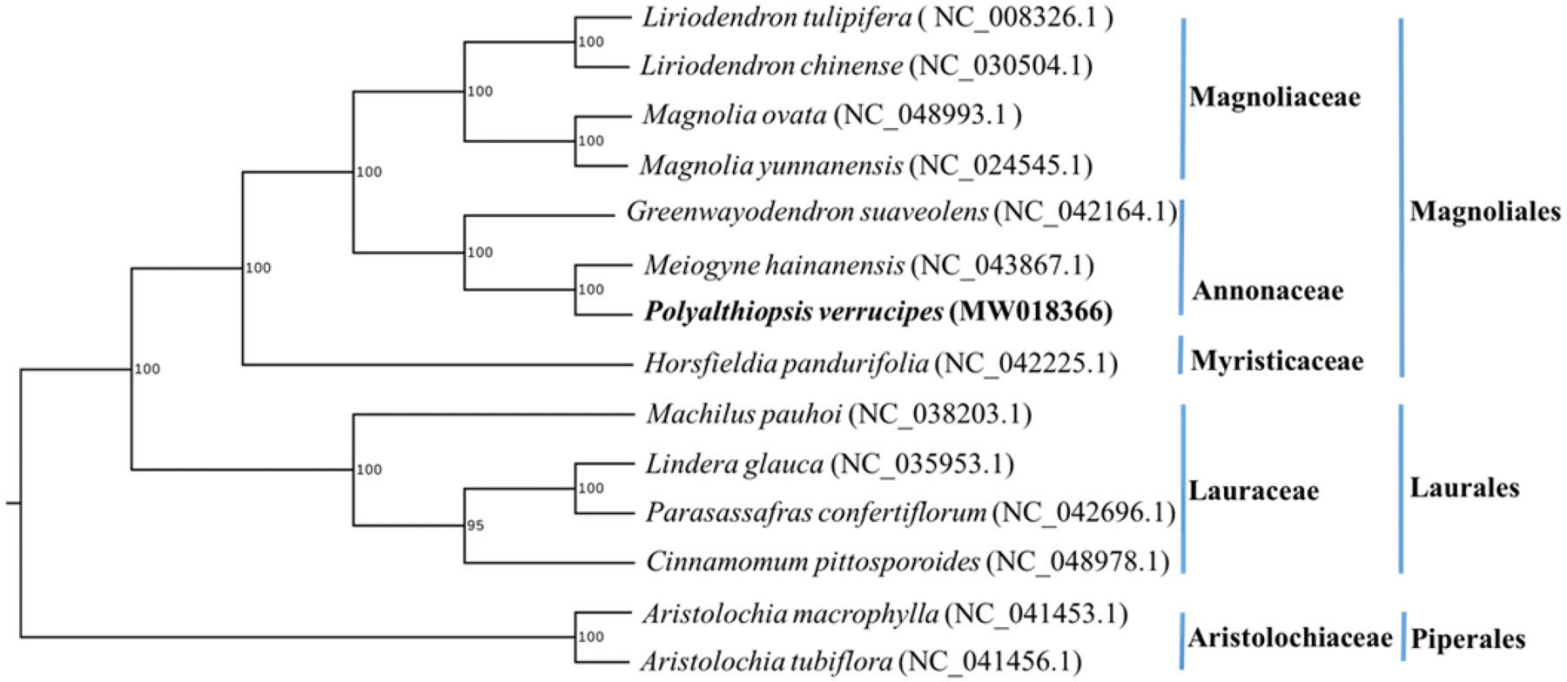
Maximum-likelihood tree of *P. verrucipes* and related species based on whole chloroplast genome sequences. Bootstrap support values (based on 1000 replicates) are shown next to the nodes.

## Data Availability

The data that support the findings of this study are available in GenBank (https://www.ncbi.nlm.nih.gov/) under the accession number MW018366.
